# Key Challenges and Opportunities for Cloud Technology in Health Care: Semistructured Interview Study

**DOI:** 10.2196/31246

**Published:** 2022-01-06

**Authors:** Kathrin Cresswell, Andrés Domínguez Hernández, Robin Williams, Aziz Sheikh

**Affiliations:** 1 Usher Institute The University of Edinburgh Edinburgh United Kingdom; 2 Department of Computer Science University of Bristol Bristol United Kingdom; 3 Institute for the Study of Science, Technology and Innovation The University of Edinburgh Edinburgh United Kingdom

**Keywords:** cloud technology, qualitative, adoption, implementation, digital health, data processing, health care, risk assessment, user engagement

## Abstract

**Background:**

The use of cloud computing (involving storage and processing of data on the internet) in health care has increasingly been highlighted as having great potential in facilitating data-driven innovations. Although some provider organizations are reaping the benefits of using cloud providers to store and process their data, others are lagging behind.

**Objective:**

We aim to explore the existing challenges and barriers to the use of cloud computing in health care settings and investigate how perceived risks can be addressed.

**Methods:**

We conducted a qualitative case study of cloud computing in health care settings, interviewing a range of individuals with perspectives on supply, implementation, adoption, and integration of cloud technology. Data were collected through a series of in-depth semistructured interviews exploring current applications, implementation approaches, challenges encountered, and visions for the future. The interviews were transcribed and thematically analyzed using NVivo 12 (QSR International). We coded the data based on a sociotechnical coding framework developed in related work.

**Results:**

We interviewed 23 individuals between September 2020 and November 2020, including professionals working across major cloud providers, health care provider organizations, innovators, small and medium-sized software vendors, and academic institutions. The participants were united by a common vision of a cloud-enabled ecosystem of applications and by drivers surrounding data-driven innovation. The identified barriers to progress included the cost of data migration and skill gaps to implement cloud technologies within provider organizations, the cultural shift required to move to externally hosted services, a lack of user pull as many benefits were not visible to those providing frontline care, and a lack of interoperability standards and central regulations.

**Conclusions:**

Implementations need to be viewed as a digitally enabled transformation of services, driven by skill development, organizational change management, and user engagement, to facilitate the implementation and exploitation of cloud-based infrastructures and to maximize returns on investment.

## Introduction

### Background

There is now an international drive toward digitally enabled, data-driven transformation of health care services, with health systems seeking to optimize work processes; improve the quality, safety, and efficiency of care; and reduce costs [1,2]. Health care typically relies on a web of complex information infrastructures that lack integration and interoperability, which contributes to fragmented service provision [3]. Such infrastructures may range from systems allowing data analysis within individual organizations to advanced cloud-based systems facilitating cross-organizational data-driven analysis [4].

Although the origins of cloud technology can be traced back to the 1960s, the term *cloud computing* has only emerged in this millennium [5]. It essentially involves delegating storage and processing of data to third-party organizations accessed via the internet rather than hosting them on an organization’s own computers. In doing so, cloud-based technologies can provide access to sophisticated large-scale technological infrastructures and advanced analytics services with the scope to rapidly scale up to meet peaks of demand [6]. Cloud product types differ in the degree of vendor and organizational control and can be public (shared across organizations), private (shared within organizations), or hybrid (a combination of both where on-premise infrastructure is combined with a public cloud). Hybrid clouds are increasingly popular as they not only allow access to public cloud infrastructure capacity but also maximize the use of on-premise solutions and therefore are a middle ground option for organizations with significant installed information technology (IT) capacity [7].

Textbox 1 summarizes the most common cloud products used in health care settings.

**Most common cloud products used in health care settings.**

**Common cloud products**

- Software as a service, where a cloud provider hosts software services that user organizations can access on the web (eg, a cloud-based electronic health record such as Athenahealth)
- Platform as a service, where providers make development tools available to the user via the cloud (eg, Microsoft Azure)
- Infrastructure as a service, where the service provider supplies cloud-based infrastructure components to the client, such as storage, servers, and networks (eg, Virtustream Enterprise Cloud)

Although cloud computing has transformed many industries (eg, entertainment and financial services) [8], its use in health care remains limited. There are some exceptions of promising developments in advanced health care systems that are now reaping the benefits (Textbox 2) [9]. The advantages of the cloud have been particularly visible in the wake of the COVID-19 pandemic, which has called for rapid deployment and cross-organizational integration of services as well as large-scale real-time data analytics [10].

**Examples of advanced health care systems that have implemented cloud technology.**

**Examples of advanced health care systems that have implemented cloud technology**

- The Shulan Health Management Group (China) implemented Amazon Web Services to host their “homegrown” system [11].
- The University of California, Los Angeles Health (United States) implemented Microsoft Azure for data processing and for integrating electronic health record data and data from other sources [12].
- The Mayo Clinic (United States) announced a strategic partnership with Google Cloud in 2019 [13].

However, despite some international governmental efforts to promote *cloud first* policies that foster the use of public cloud offerings in technology procurement [14,15], there are still significant points of friction in the adoption of cloud-based services. Some of these include concerns about security; fears of potential legal disputes between service providers and organizations; and issues surrounding vendor lock-in, privacy, ethics, and data ownership [16-20].

### Objectives

In this study, we seek to understand how current opportunities in data-driven innovation facilitated by cloud computing could be positively harnessed in health care settings while minimizing perceived or actual risks.

## Methods

### Overview

We conducted a qualitative study between September 2020 and November 2020 using semistructured interviews sampling cloud providers, system implementers, software vendors, customers, and health informatics academics to gain an in-depth understanding of the evolving cloud ecosystem. It is important to keep in mind that data collection took place in the midst of the global COVID-19 pandemic and in the context of ongoing deliberations on the potential uses of cloud technology to address emerging urgent pandemic-related challenges. Discussions were strongly influenced by this topic.

### Ethical Approval

We obtained ethical approval before the start of the study from the Usher Institute Research Ethics Group at the University of Edinburgh. Participants were provided with a consent form and an information sheet describing the study aims, procedures, and data management practices before participating in the study. They were given at least 48 hours to consider whether they agreed to participate and provided written informed consent. We informed the participants that they were free to withdraw at any time and that their responses would be anonymized during the analysis, removing names and places that could lead to identification of individuals.

### Recruitment of Participants

We purposefully sampled stakeholders with perspectives on the topic of implementation, adoption, and optimization of cloud technology in health care settings [21]. Our aim was to gain a broad overview of different perspectives to understand the challenges and opportunities around cloud technology in health care settings and draw lessons that could inform future strategies for decision makers. In doing so, we specifically targeted individuals working across technology implementation, operations, design, research, and innovation within a range of organizations. We identified and recruited participants through our existing networks and communication channels as well as Google and LinkedIn searches using keywords related to the cloud and eHealth (eg, *digital health*, *digital transformation of health*, *cloud computing*, and *cloud first*). We complemented this strategy through snowball sampling by asking participants for recommendations of further interviewees. We aimed for variability in terms of geographical location (not including low- and middle-income countries as existing information infrastructures and challenges in these countries are likely to vary significantly), organizational function, area of expertise, and gender. Participants were selected based on their relationship with cloud technology in health care, both from the supply (cloud and software vendors) and demand (health care providers) sides. This included those who had experiences and opinions on the topic through experience of developing cloud solutions and cloud-enabled software, implementing and operating systems, or researching cloud technology.

### Data Collection

ADH, a researcher with a background in science and technology studies and theoretical foundations surrounding information infrastructures, conducted all interviews via videoconference call software (Microsoft Teams). Interviews took the format of a *conversation with a purpose* where participants were encouraged to discuss issues important to them. ADH and KC (a social scientist with a background in sociotechnical theory) met periodically throughout the data collection process to discuss emerging findings and modify key lines of inquiry.

The interviews ranged in duration from 40 to 70 minutes. There were 20 one-to-one interviews and 1 group interview with 3 participants. Where participants asked for a group interview, we accommodated this request as it was more convenient for the participants and allowed us to gain insights into their complementary perspectives simultaneously. Although questions were tailored to individual roles and modified in line with emerging findings, we followed a topic guide exploring the state of cloud-enabled digital transformation in health care; views on barriers to realizing the potential benefits, risks, and areas of concern; and suggestions on how to address them (Textbox 3). During this process, the interviewer incorporated emerging themes across various interviewees and explored the tensions and differences in viewpoints in detail. We stopped collecting data when no new themes emerged during the concurrent analysis [22].

**Topic guide.**

**Topic guide**

- Interviewee’s background, current position, and description of the organization
- Overview of the cloud ecosystem, stakeholders, and existing offerings
- Implications of cloud adoption (cultural, organizational, operational, and financial adoption around digital transformation processes)
- Promising and concrete use cases of cloud technology in health care
- Challenges, risks, and hindrances for innovation in the cloud
- Distinctive challenges of health care compared with other industries and sectors
- Concerns about privacy, security, data ownership, and ethics
- State of affairs and challenges in terms of integration and interoperability between cloud platforms
- Role of the government
- Future outlook (5-10 years) of the cloud in health care

### Data Analysis

The interviews were transcribed using an external professional service and subjected to thematic analysis [23]. ADH verified the interview transcripts by listening to the audio recordings and correcting any inaccuracies before analysis.

We used a mixture of deductive and inductive thematic coding [24]. We added the transcripts to an NVivo (QSR International) version 12 project and theme coded them using a sociotechnical coding framework developed by the research team [25]. This framework highlights how different technological and social dimensions interrelate and how different perspectives shape aspects of the implementation and adoption of new technologies (Textbox 4). In addition, identified themes that did not fit the analytical framework were included in new categories.

**Dimensions used in the Technology, People, Organizations, and Macroenvironmental factors coding framework.**

**Dimensions used**

- Technology (the technological properties of the system and the surrounding infrastructure)
- People (how various stakeholders use technology, including their expectations and experiences)
- Organizations (how organizations implement technology and how this shapes use)
- Macroenvironmental factors (how political and economic factors and markets shape technology development, use, implementation, and optimization)

ADH performed the first round of coding, periodically discussing emerging findings with KC. KC then re-examined the codes, resulting in minor changes to node titles and summarized the results in a narrative format. As part of our reflexive process, we identified how our previous experiences, assumptions, and preconceptions bore on the interpretation and coding of the data. In doing so, we discussed emerging findings within the research team to identify the relevance of themes within the Technology, People, Organizations, and Macroenvironmental factors (TPOM) framework as well as the need for new categories. We focused on examining converging and diverging perspectives, the interplay of technological and social dimensions, and the tensions and trade-offs emerging in the progress of cloud technology implementation, adoption, and optimization in health care settings.

## Results

### Overview

We interviewed 23 individuals (Table 1), including professionals working across major cloud providers, health care provider organizations, innovators, small and medium-sized software vendors, and academic institutions.

**Table 1:** Characteristics of the participants.

Participant number	Gender	Location	Occupation	Organization
1	Female	United States	Executive	Cloud vendor
2	Male	United Kingdom	Executive	Software vendor
3	Male	United Kingdom	Executive	Health care provider
4	Male	United Kingdom	Executive	Software vendor
5	Male	United Kingdom	Operations	Cloud vendor
6	Male	United Kingdom	Operations	Health care provider
7	Male	United Kingdom	Executive	Software vendor
8	Male	United Kingdom	Operations	Health care provider
9	Male	France	Operations	Cloud vendor
10	Male	United Kingdom	Operations	Cloud vendor
11	Male	United Kingdom	Academic	Health care provider
12	Female	United States	Operations	Cloud vendor
13	Female	United Kingdom	Operations	Cloud vendor
14	Female	Finland	Academic	Research
15	Male	United Kingdom	Operations	Software vendor
16	Female	United Kingdom	Executive	Research
17	Male	United Kingdom	Executive	Health care provider
18	Male	United Kingdom	Operations	Software vendor
19	Male	United Kingdom	Executive	Software vendor
20	Male	United Kingdom	Executive	Software vendor
21	Male	United Kingdom	Implementer	Health care provider
22	Female	United Kingdom	Operations	Cloud vendor
23	Male	United States	Executive	Cloud vendor

We produced 40 codes within the following four thematic areas: organizational context, social-human factors, technological factors, and wider macroenvironmental factors. The researchers then discussed the codebook and identified 4 salient challenges that were common across different interviewee backgrounds and affiliations. These were (1) drivers and perceived benefits associated with cloud technology in health care; (2) organizational and technological barriers limiting cost-effective use of cloud functionality; (3) infrastructural changes not immediately visible to frontline users, resulting in lack of clinical pull; and (4) visions of the future cloud vendor ecosystem.

**Figure 1:**
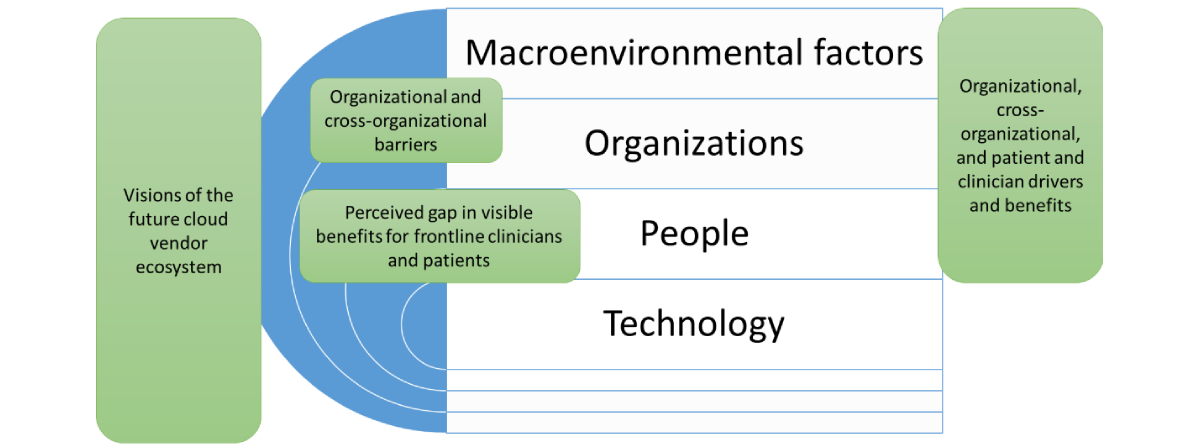
Overview of findings mapped onto the Technology, People, Organizations, and Macroenvironmental factors framework.

Figure 1 illustrates how these emerging themes map onto the TPOM framework. As illustrated, the new emerging overarching categories related to cross-cutting issues spanning more than one TPOM dimension.

### Drivers and Perceived Benefits of Cloud Technology in Health Care

The participants described various uses of cloud functionality, including scheduling software, videoconferencing, call center management, imaging analysis, and patient data analytics. On the basis of the most frequently discussed uses, we identified three salient categories: (1) organizational dimensions (eg, remote and collaborative working at scale, modeling algorithms surrounding predictive analytics, organizational analytics, and automation), (2) patient- and clinician-facing (eg, remote working, chatbots, and community outreach functionality), and (3) cross-organizational and regional (eg, data analytics surrounding particular disease areas for population health management and research).

We observed overall positive attitudes among the participants in relation to how cloud computing helped harness the value of data-driven innovation at scale. The adoption of cloud technology was perceived to be driven by existing issues faced by the sector, particularly concerning limited resources, access to and delivery of care, administrative workloads, and availability of critical services. Positive attitudes were particularly salient among system implementers, who saw immediate gains through secondary uses of data and tackling some of the most pressing challenges for health care posed by the COVID-19 pandemic. Here, cloud technology facilitated the deployment of solutions at speed without the need to purchase additional hardware:

[Cloud] enabled responsiveness...and throughout COVID, that’s what that’s been about. And it’s removed one challenge [of] getting hold of hardware, getting it set up and all the rest. So, it’s made us more responsive, it’s made us quicker to adapt...the forcing function was COVID, and cloud’s helped us have a faster response.

Participant 2, male, software vendor, United Kingdom

Other key benefits associated with cloud technology mentioned by the participants included cost-effective management and storage of data at scale combined with ready access to advanced computing capabilities and tools, such as machine learning (ML) and natural language processing:

And one of the greatest things about this now is machine learning and AI [artificial intelligence]...it hasn’t been up until recently when [vendor] fully put a heavy effort over the last five or six years about democratizing access to these tools at scale, because you’re not only interested in building one or two models, you’re interested in building hundreds, thousands, tens of thousands of these models.

Participant 23, male, cloud vendor, United States

### Organizational and Technological Barriers Limiting Cost-effective Use of Cloud Functionality

Although benefits associated with cloud technology were realized in many organizations that the participants worked with, they also discussed how these might not be representative of the wider health care landscape. Barriers manifested differently depending on the existing organizational and technological capabilities. Data migration and acquisition costs were mentioned by many interviewees from both the supply and demand sides. Cloud technology posed fewer barriers to organizations with few installed on-premise systems that sought to either implement new *pure* cloud-based solutions or rely on a *software as a service* business model. On the contrary, organizations with relatively mature digital infrastructures and legacy systems faced hurdles to transition to cloud only solutions as they had to integrate existing systems and replace core infrastructures. Existing legacy systems were based on proprietary data structures and workflows, meaning that they could not simply be imported into the cloud. Instead, these organizations were more amenable to hybrid cloud solutions that relied on *infrastructure as a service* implementations:

The problem is, the cost of transition, if you’re talking about your patient administration system or your electronic health record, which is often the core bit of software in your health care organization, if they want to switch that out, it is a huge job, which is massively expensive and massively risky to do.

Participant 4, male, software vendor, United Kingdom

In addition, implementers in particular raised the barriers associated with the need to change their cost structure with cloud technologies from capital up-front investments to a revenue model with recurring costs. This was perceived to be particularly problematic during the transitional period, when organizations were often running and paying for parallel systems:

In the short-term, you are inevitably paying more for the move towards cloud because you haven’t necessarily got rid of all of that other infrastructure as you make that transition. So, you’re now starting to pay for a revenue cost for your new cloud platform, but you’ve still got all of the cost of that other physical environment until you’re able to decommission.

Participant 16, female, research, United Kingdom

Barriers not only related to cost but also to the organizational capabilities to adopt cloud solutions. Here, a lack of existing knowledge and skills in organizations to deploy and exploit cloud functionality was an important rate-limiting step. For instance, organizations frequently lacked implementation and migration skills:

In order to move things securely to the cloud either to implement brand new or to do a migration, you know, you need to have a certain degree of skill, knowledge, capability in order to do that...

Participant 4, male, software vendor, United Kingdom

Existing technical skills and capabilities also played an important role in maximizing the benefits of cloud functionality once it was implemented. Here, participants stated that many health care organizations lacked the knowledge and skills needed to work with advanced large-scale data analytics and therefore struggled to optimize the use of cloud infrastructures through artificial intelligence and ML:

There’s a step that still needs to happen in the healthcare space, which is around just understanding what the analytics is.

Participant 19, male, software vendor, United Kingdom

Other barriers inhibiting uptake of cloud technologies in health care organizations included the changes in organizational culture required to transition to externally hosted systems and new modalities of accessing critical services. This was seen as particularly problematic for a risk-averse sector such as health care. For example, some participants mentioned that organizations that were skeptical about implementing cloud technology feared a loss of control if they migrated their IT systems to external service providers. In addition, there was apprehension about the reliability of the cloud and telecommunication infrastructure to deliver critical services, which manifested in the perceived need to fall back on on-premise IT services as contingency measures for critical services:

Traditionally, IT departments in [provider organizations], you have your server, you have your software on it, and they manage that. It makes them slightly uneasy if it’s out there in a cloud and it’s not something that they have control of.

Participant 4, male, software vendor, United Kingdom

Others stated that moving to cloud technology threatened established organizational hierarchies, particularly when sharing data across organizations. Health care settings were often not used to working across organizational boundaries. Cloud services challenged the traditional conception of organizations as autonomous entities and posed dilemmas in relation to information governance:

It’s about [organizations] having to give up something to be part of a bigger collaboration.

Participant 21, male, health care provider, United Kingdom

### Infrastructural Changes Not Immediately Visible to Frontline Users, Resulting in Lack of Clinical Pull

Although the organizational benefits of a wide range of cloud-based functions were visible and the case for organizational process and workload improvements could be made relatively easily by suppliers and system implementers, there was a perceived gap in visible benefits for frontline clinicians and patients. This presented a key barrier to the wider uptake of some cloud-based services as end users need to be on board for organizational changes to be implemented effectively:

An organizational imperative has to pass the challenge of the clinicians’ view of what is important and vice versa. The clinicians’ view of what is important has to pass the challenge of the gatekeepers in terms of organization, of funding, of development, service development, building development.

Participant 18, male, software vendor, United Kingdom

The underlying issue was the invisibility of digital infrastructures for those at the frontline, who mainly experienced benefits through the exploitation and optimization of these infrastructures once they were in place:

The people who are going to be using the technology, the people who are going to be using the insights from the analytics, the people who will be experiencing the change in process, they are almost don’t really, it might sound harsh but...in the heat of the moment they almost don’t really care about is it cloud enabled? What is the infrastructure? What’s going on? Like, they just want the front end to work.

Participant 19, male, software vendor, United Kingdom

This lack of immediately visible benefits for end users combined with concerns surrounding privacy and security and the handling of sensitive data led to a lack of active user pull for cloud technologies in health care. It also presented challenges for suppliers as they had to satisfy a range of demands surrounding not only business processes but also clinical utility:

There’s some particular challenges, how do you deal with the privacy aspects of the data and satisfy the concerns that data contributors and data custodians have, and then how do you accommodate for this enormous diversity within the user community in terms of how they use data and importantly how they get beyond very simple table analytics views of data into something that is more problematic, and how do you find a way for those outputs, those research outputs, to make their way back into clinical utility.

Participant 7, male, software vendor, United Kingdom

Despite these uneven perceptions, we also observed that during the COVID-19 crisis, clinical benefits and experiences of cloud technology became more common and thus immediately visible as remote consultations, remote working, data storage, and automation (eg, through chatbots) increasingly became a necessity:

Overnight we did see this huge uptick in the amount of telehealth, and that was only possible because of cloud there to support it.

Participant 22, female, cloud vendor, United States

### Visions of the Future Cloud Vendor Ecosystem

Innovators, implementers, academics, and cloud vendors agreed on a vision characterized by a hybrid cloud-enabled ecosystem of applications where software suppliers rely on a combination of on-premise systems and cloud integration with a large cloud provider. For software suppliers, integrating with a cloud platform meant that they could quickly and cost-effectively scale up and scale down their products as required. This, in turn, was perceived to translate to lower risk and more efficient costing for health providers surrounding the trialing of new services:

For us, the main use cases are around working with a platform that allows us to quickly and cheaply get our product out into market...we don’t need to invest huge amounts of time and people in developing things that are already out there...We can manage and maintain one environment, rather than having to think about how do you easily deploy and support, maintain, you know, 10, 20, 100 different customers, and the intricacies of deploying our app at every single customer site. We only have to think about one location.

Participant 20, male, software vendor, United Kingdom

However, the participants (in particular, implementers and software developers) also flagged the challenges and risks in terms of interoperability between different platform providers and integration between software vendors and cloud vendors. Innovators and system implementers voiced their expectations for interoperability standards and for cloud providers to open up application programming interfaces. However, opening up application programming interfaces and standardizing key functions was not always in line with legacy providers’ commercial interests, which were typically based on retaining users within their platforms. Therefore, innovators in the software industry and implementers within health care organizations called for national regulations specifying interoperability standards to avoid vendor lock-in as this would allow for integration between systems and improve data portability. A lack of interoperability standards was viewed as inhibiting the development of a vibrant cloud ecosystem:

These regulatory bodies inside each of the governments would say the same thing, because that is the way to drive adoption of new technologies, forcing the new adoption, not rewriting everything, that’s out of the question, but forcing for the benefit of all. I think this is how you’re going to be having a government that is strong on that.

Participant 9, male, cloud vendor, Europe

## Discussion

### Principal Findings

Although the participants perceived clear drivers for the use of cloud technology in health care settings, particularly in relation to collaboration and workload efficiencies, barriers to progress included data migration costs and skill gaps within health care organizations to support implementation. This was exacerbated by the perceived cultural shift required to move to externally hosted services, challenging entrenched organizational ways of working and the need to reorganize existing cost structures. Frontline users, particularly those lacking technical expertise, were not directly concerned with the benefits associated with cloud-based infrastructures, which resulted in a lack of user pull in organizations seeking to change their technological infrastructures. However, the pressures of the COVID-19 pandemic and the stronger need for remote working arrangements made various critical cloud services visible. Central regulations and mandated interoperability standards were viewed as a key priority to foster innovation and reduce the risk of vendor lock-in.

### Integration of Findings With the Current Literature

Our study confirms findings in other sectors that highlight that, despite the potential benefits, the move to cloud-based technologies in organizations necessitates cultural shifts from established ways of working and administering systems [26]. Therefore, it needs to be viewed as a complex sociotechnical transformation process, requiring not only technological but also socio-organizational changes to maximize the potential of cloud technologies [27]. Here, changes in organizational business models and technological infrastructures associated with cloud technology are likely to affect existing ways of working and organizational functioning as a whole [28]. Therefore, a key area of focus needs to be the effective integration and embedding of new infrastructures with the *installed base* of existing technologies and socio-organizational structures and practices [4]. Barriers associated with data migration to cloud-based solutions are well documented in the literature [29], but our work also points to differences between digitally mature organizations with established installed technological systems (requiring more fundamental changes to the installed technological base) and those organizations that do not have established technological infrastructures, where data migration is likely to be less of an issue.

There is an asymmetry in the way system implementers, clinicians, and patients perceive and understand the benefits of the cloud, particularly when it comes to advanced functions such as ML and data-driven functionalities, which results in a lack of strong user pull [30]. User pull to implement cloud technologies within organizations is critical, especially in public service sectors [31]. Here, user attitudes and expectations toward technology can have a direct impact on adoption patterns [32]. A lack of perceived direct benefits as well as skepticism and concerns (most notably, perceived security, trust, and privacy issues) can result in negative attitudes toward a technology and lead to abandonment [33]. There are now growing calls for transparency and accountability of how personal data are used within cloud-based systems without compromising privacy and security [34]. Medical research is a key area where clinical data are considered immensely valuable but where handling of sensitive data is of utmost importance. This issue intersects not only with privacy and security but also with growing interest across industry and academia on trustworthy, fair, and ethical use of big data and algorithmic technologies [20,35,36]. Therefore, it is critical for organizations promoting the use of cloud technology to place emphasis on active engagement with users and rigorously engage with debates about privacy, ethics, and security taking place in academic and public forums [37].

The move to cloud technologies in health care presents a disruptive innovation for the market [30,38], which inevitably results in tensions and trade-offs between conflicting agendas and interests. In this study, we observed that points of friction related to the integration of different building blocks and interoperability between competing platforms. These challenges resonate with previous studies in information systems, which highlight ongoing tensions between requirements for standardization and the flexible and cost-effective operation of systems [39-41].

### Implications for Policy, Practice, and Research

Among the key challenges voiced by our informants were the lack of installed capacity and technical skills, the cost of migration, and the need for investment restructuring. As a result, organizations that still rely on on-premise IT infrastructure and software see hybrid cloud solutions as a way forward. There is now a need to support the development of such hybrid structures and map potential integration and migration pathways to help implementing organizations envisage new information infrastructure constellations. This needs to be supported by active efforts to address the existing skill gap in cloud computing and digital transformation expertise in the health sector [42]. This will also help ensure that advanced cloud functions such as ML are effectively exploited.

Strategic decision makers need to recognize the need to view the implementation of cloud-based systems as a major digital transformation of services to promote cloud first policy in health care settings. Therefore, implementations need to be supported not only by technological capability but also by change management expertise and continuous stakeholder engagement.

Our work highlights divergent views and expectations among various stakeholder groups in relation to interoperability. These are highly contingent upon political-economic contexts as interoperability standards are not always centrally mandated across countries. Innovators and system implementers in particular raised the need to regulate the emerging cloud ecosystem through the development of interoperability standards. Adding to the risk of developing solutions for a particular vendor is poor integration between competing platforms. A clear policy recommendation to address this challenge is the central mandate for interoperability standards, with the United States being a case for reference, but these need to be flexible to respond to emerging needs and other disruptive innovations that are likely to emerge. Of central importance will be the need for trustworthy entities and tools for responsible use of sensitive data, developing mechanisms for ensuring ethical and transparent use for medical research without compromising patients’ privacy and integrity.

### Strengths and Limitations

We gained insights into the opportunities and challenges in the emerging area of cloud technology implementation in health care settings by consulting a range of perspectives. We deliberately sampled implementers, customers, academics, and vendors to explore experiences and insights from a range of settings. However, this may have been at the expense of breadth. For example, consumer and customer perspectives were underrepresented in our sample, and we did not consult the range of immediate frontline users of technologies or legal and privacy experts. Our sample also consisted mainly of cloud enthusiasts. Nevertheless, our study points to various user-facing issues such as adoption, use, concerns, and invisibility of functions, which we assessed indirectly through respondents working in close contact with users. Further empirical work with clinicians, lawyers, and privacy experts arises as a pertinent avenue of research.

Our themes provide a helpful guide for conducting future in-depth work as we have illustrated an overview of tensions. In addition, we would also have liked a broader representation of international settings (as 18/23, 78% of participants in our sample were based in the United Kingdom). Our current sample consisted mainly of participants from North America and Europe (France, Finland, and the United Kingdom). Future work should build on our findings seeking to explore how different geographies, including low- and middle-income countries, have approached the area and how challenges vary across different core infrastructures, levels of digital maturity, and health system organization.

### Conclusions

Although cloud technologies promise to deliver a range of technical capabilities, they are unevenly applied across health care settings depending on organizational contexts and existing infrastructures. In the wake of the pandemic, cloud technologies have become vital to support everyday collaboration for clinicians, remote health delivery, and other operational functions, which has considerably driven the adoption of the cloud. Going forward, cloud implementation needs to be viewed as disruptive organizational change initiatives facilitated by national initiatives to promote interoperability for a vibrant cloud ecosystem. Areas that may lend themselves to such work may include patient-facing technologies, where cloud providers are already established, and health and social care integration, where limited existing health information infrastructures may reduce barriers associated with integration or migration. This will also need to involve engaging in public discourse about cost, risk, and trust (or lack thereof) in cloud platforms regarding the handling of sensitive data, privacy, security, and ethics.

## Abbreviations

IT: information technology
ML: machine learning
TPOM: Technology, People, Organizations, and Macroenvironmental factors
